# Carrying Position-Independent Ensemble Machine Learning Step-Counting Algorithm for Smartphones

**DOI:** 10.3390/s22103736

**Published:** 2022-05-13

**Authors:** Zihan Song, Hye-Jin Park, Ngeemasara Thapa, Ja-Gyeong Yang, Kenji Harada, Sangyoon Lee, Hiroyuki Shimada, Hyuntae Park, Byung-Kwon Park

**Affiliations:** 1 Department of Management Information Systems, Graduate School, Dong-A University, Busan 49315, Korea; s2403353662@gmail.com; 2 Department of Health Sciences, Graduate School, Dong-A University, Busan 49315, Korea; hjpark3987@gmail.com (H.-J.P.); ngeemasara@gmail.com (N.T.); sky940702@naver.com (J.-G.Y.); 3 Department of Preventive Gerontology, National Center for Geriatrics and Gerontology, Obu 474-8511, Japan; harada-k@ncgg.go.jp (K.H.); sylee@ncgg.go.jp (S.L.); shimada@ncgg.go.jp (H.S.)

**Keywords:** pedometer, step-count algorithm, smartphone, machine learning, wearable position, acceleration signal processing

## Abstract

Current step-count estimation techniques use either an accelerometer or gyroscope sensors to calculate the number of steps. However, because of smartphones unfixed placement and direction, their accuracy is insufficient. It is necessary to consider the impact of the carrying position on the accuracy of the pedometer algorithm, because of people carry their smartphones in various positions. Therefore, this study proposes a carrying-position independent ensemble step-counting algorithm suitable for unconstrained smartphones in different carrying positions. The proposed ensemble algorithm comprises a classification algorithm that identifies the carrying position of the smartphone, and a regression algorithm that considers the identified carrying position and calculates the number of steps. Furthermore, a data acquisition system that collects (i) label data in the form of the number of steps estimated from the Force Sensitive Resistor (FSR) sensors, and (ii) input data in the form of the three-axis acceleration data obtained from the smartphones is also proposed. The obtained data were used to allow the machine learning algorithms to fit the signal features of the different carrying positions. The reliability of the proposed ensemble algorithms, comprising a random forest classifier and a regression model, was comparatively evaluated with a commercial pedometer application. The results indicated that the proposed ensemble algorithm provides higher accuracy, ranging from 98.1% to 98.8%, at self-paced walking speed than the commercial pedometer application, and the machine learning-based ensemble algorithms can effectively and accurately predict step counts under different smart phone carrying positions.

## 1. Introduction

Step-count algorithms are valuable for various applications, some of which are- physical activity measurements [1,2,3], pedestrian dead reckoning [4], and physical rehabilitation [5,6,7]. Recently, smartphone pedometer applications have gained more attention due to their cost-effectiveness compared with commercial pedometer devices. According to statistics that surveyed the global smartphone penetration rate, the global smartphone penetration rate is estimated to have reached 78.1% in 2020 [8]. This implies that most people can monitor their steps using a smartphone without additional devices. However, smartphone pedometer applications have a notable drawback, i.e., insufficient and unstable step-count accuracy, caused by the various carrying positions of smartphones [9,10].

To resolve this issue, several step-count algorithms [11,12,13,14] have been proposed in the last few years. In a study of a smartphone-based step-count algorithm that could mitigate false step counts caused by random motions during phone handling, a step-count accuracy of 98.7% was reported [14]. However, the algorithm uses an accelerometer, a gyroscope, and magnetometer sensors, which are significantly power-consuming [15]. Another study [10] used a principal component analysis algorithm to reduce the dimension of the gait feature and a random forest classification algorithm to identify the carrying position of smartphones and reported a highly improved step-count accuracy. Although the result was obtained on small datasets (approximately 250 steps in total), it shows that the accuracy of step counts can be improved after identifying the carrying position.

The label dataset size limits machine learning-based step-count algorithms in most studies. These studies collected label (number of steps) data using a mechanical counter or by counting steps from a video [11,12,13,16]. These methods are cost-efficient and straightforward but not feasible for recording data continuously or accurately in long-term experiments. Generally, the more training data a machine learning-based algorithm has available, the better the performance. Hence, a better method is needed to collect label data over a prolonged period to provide more training data to the machine learning-based algorithms to improve step-count accuracy. The step-count method based on insoles integrated with pressure sensors can solve this problem because of their high step-count accuracy (96% to 100%) and convenience [17,18,19]. These insoles mainly use Force Sensitive Resistor (FSR) sensors to measure the ground reaction force dynamically [20], which is suitable for the stable measurement of gait cycles [21], even on uneven surfaces such as stairs [22]. However, no commercial device can simultaneously record plantar pressure and smartphone acceleration data. Although it is possible to use a separate pressure insole and smartphone acceleration recording application, the following issues exist:

1. A system to keep the clock of the pressure insole consistent with the smartphone’s clock.
2. Shut down of the data recording application that has been running in the background for too long, resulting in incomplete data recording.

Therefore, it is challenging to build a stable system that combines the pressure data obtained from the FSR sensors with the acceleration data obtained from smartphones for training the machine learning-based algorithms.

In this study, we first developed a data-collection system for the problem of hard-to-obtain label data. Then we collected a large amount of data to train step-counting models by labeling the number of steps detected in the pressure peaks obtained from the plantar pressure sensor. To the best of our knowledge, no such study has been done before. Moreover, since the different carrying positions of the smartphone result in different patterns of acceleration data, a classification algorithm was used to identify the carrying position of the smartphones before a regression algorithm was used to count the steps.

The proposed algorithm was compared with a commercial pedometer application under self-paced walking conditions to verify its reliability and performance. Six participants participated in the experiment. The results show that this method dramatically improves the accuracy and stability of the step-counting model. In summary, our work makes the following contributions:

1. A plantar pressure and smartphone acceleration data-collection system.
2. Data processing methods to convert raw plantar pressure data and smartphone acceleration data into datasets usable by machine learning models.
3. A carrying position-independent step-counting algorithm, which detects the position through a classification model, and then uses the corresponding regression model to count steps.
4. An evaluation of the proposed step-counting algorithm based on extensive samples collected from six participants, and a comparison of the performance of our approach to a commercial pedometer.

The rest of the paper is organized as follows: In Section 2, we describe the data-collection system, data pre-processing methods, and data labeling algorithms. Then, we describe the model building process. In Section 3, we describe the experimental procedure and the structure of the dataset, and we then evaluate the performance of the proposed algorithms. In Section 4, we discuss the study results, and the conclusions are given in Section 5.

## 2. Methods

### 2.1. Data Acquisition

**Figure 1:**
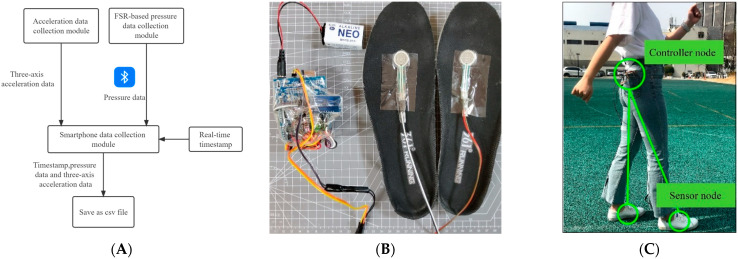
(**A**) The FSR-based pressure data-collection module measures the number of steps by collecting plantar pressure data. The IMU data acquisition module uses an Android API to collect the three-axis acceleration data. After the data are collected, a real-time timestamp is added to align the acceleration data and the pressure data’s time. (**B**) The FSR-based pressure collection module. (**C**) The wearing position of the FSR-based pressure collection module.

A data-collection system was developed to collect the acceleration data from smartphones and plantar pressure data. In contrast to existing FSR-based smart insoles (such as Footslogger [19]) and smartphone-based acceleration acquisition software (such as inertial measurement units (IMU) Logger and Sensor stream IMU), the proposed data acquisition system integrates the smartphone’s acceleration data-collection module and the FSR-based pressure collection module into a single application (Figure 1A). The two types of data are finally processed and stored on a smartphone. This solved the problem of the data acquisition system being too complicated when collecting the gait data. Data collection was performed in the following steps:

1. The smartphone connects to the Arduino board and sends the current 13-digit timestamp after the user clicks the start recording button. The Arduino board starts recording the plantar pressure data and adding the timestamp. The smartphone starts recording the acceleration data.
2. The Arduino board sends the pressure data and timestamp to the smartphone at 30 Hz.
3. After the user clicks the stop recording button, the smartphone will disconnect after the last data received from the Arduino board.

#### 2.1.1. Acceleration Data Collection Module

The acceleration data-collection module was developed through the Uni-app Framework [23], and three-axis acceleration data were obtained through the API provided by HTML5 + Plus [24]. The present data-collection module was designed to collect data at 30 Hz. This study used a smartphone with Android 8.1 (Rakuraku F-01L, Fujitsu, Japan). To avoid background operating restrictions by the Android system [25], we applied a long-term data recording function to allow for listing and self-starting in the background.

#### 2.1.2. FSR-Based Pressure Data Collection Module

The FSR-based pressure data-collection module collected plantar pressure data and transmitted them to the data-collection module in the smartphone via Bluetooth (Figure 1A). The module consists of the following three components:

- FSR sensor
  Researchers favor FSR sensors because of their simplicity, flexibility, low cost, and high durability. We used IMS-C20A FSR sensors to collect plantar pressure data. The IMS-C20A FSR sensors were circular and 20 mm in diameter. The sampling frequency of pressure data was 30 Hz. The IMS-C20A FSR sensors have a sensitivity range of 0.05 kg to 6 kg. Pressures above or below this range will hardly cause changes in resistance values. Therefore, we identified values less than 0.5 N as 0 N and greater than 60 N as 60 N. The IMS-C20A FSR sensors were placed on the insole near the toe to collect the plantar pressure data for each gait cycle (Figure 1B).
- Arduino controller with battery and Bluetooth module
  An Arduino Uno development board was used to collect the pressure data from the FSR sensors, and data were transmitted to the smartphone’s data-collection module via the Bluetooth modular. We used a model JDY-16 Bluetooth module, which is based on Bluetooth 4.2 standard; the working frequency is 2.4 GHz, the modulation mode is GFSK, the bit rate is 115,200 bps, the maximum transmission distance is 80 m, and the communication rate is 8 Kbytes per second. We placed the controller node far away from the sensor nodes and used larger-capacity batteries along with sub-components to solve the problem of insufficient power caused by real-time data collection and transmission. The device was placed in a trouser pocket (Figure 1C) for convenience, allowing the subject to change the batteries quickly.
- Data acquisition and storage components
  The data acquisition and storage components collected pressure data and three-axis acceleration data at 30 Hz, and an Android smartphone was used to save data and time stamps to match the acceleration data and pressure data. The frequency range of human walking and running is approximately 0.5–5 Hz [26,27], and according to the Nyquist criterion, the sampling frequency should be at least twice the target frequency to obtain complete information. Therefore, the sampling frequency in this study was set to 30 Hz.

### 2.2. Data Pre-Processing

This study focused on smartphones’ three-axis acceleration data and plantar pressure data. To train and fit the machine learning-based algorithms to count steps, we used a series of algorithms to pre-process the data.

First, the Signal Vector Magnitude (SVM) algorithm [28,29,30] was used to compute the magnitude from the raw three-axis acceleration data (including gravitational acceleration) to eliminate the noise caused by the changes in the smartphone’s angle. The SVM is defined as follows:

(1) $$\mathrm{SVM}=\sqrt{X^{2}+Y^{2}+Z^{2}}$$

where *X*, *Y*, and *Z* represent the components of acceleration on the x-, y-, and z-axis, respectively.

Subsequently, a Moving Average Filter (MAF) [29] was applied to eliminate noise and obtain a smooth signal from the acceleration magnitude and plantar pressure data. The MAF is defined as follows:

(2) $$A_{t}=\frac{a_{t-2}+a_{t-1}+a_{t}+a_{t+1}+a_{t+2}}{5}$$

where $a_{t}$ is the unfiltered signal at position *t*, and $A_{t}$ represents the filtered version of the signal. All four closest neighbors were assigned the same weight.

A peak detection algorithm was applied to extract the peak value of the plantar pressure data after a MAF was used. Finally, a sliding window algorithm extracted the input data and labels.

### 2.3. Data Labeling

#### 2.3.1. Plantar Pressure Data and Gait Cycle

**Figure 2:**
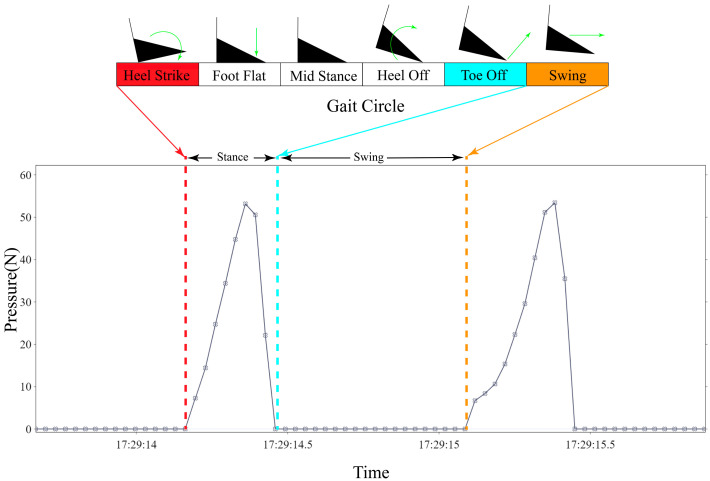
Pressure changes during two gait events at average gait speed (0.8–1.4 m/s).

A gait cycle is defined as a series of actions comprising the foot touching the ground, leaving the ground, and reaching the ground again [19]. It is a combination of phases, usually marked as (i) heel strike (HS), (ii) foot flat (FF), (iii) mid stance (MS), (iv) heel off (HO), and (v) toe off (TO) [31]. A complete gait cycle consists of a stance phase and a swing phase, which can be detected by changes in plantar pressure [32,33] (Figure 2). Zero pressure indicates that the foot is not in contact with the ground (swing), whereas a non-zero value represents the stance stage in which the foot is in contact with the ground. Consequently, the number of steps can be calculated by counting the number of pressure changes from zero to non-zero.

#### 2.3.2. Threshold-Based Peak Detection Algorithm for Pressure Data

**Figure 3:**
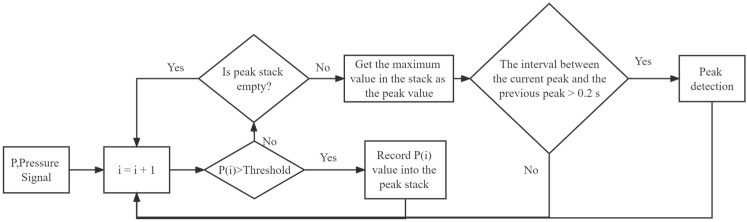
The flow diagram of the threshold-based peak detection algorithm.

**Figure 4:**
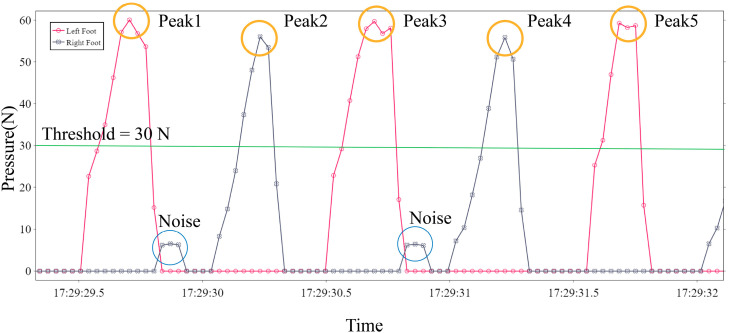
Effect graph of the threshold-based peak detection algorithm.

The gait cycle can be monitored by distinguishing between zero and non-zero values of plantar pressure. However, the pressure sensor placed on the insole produces noise caused by uneven heat distribution or by the foot contact when swinging [19]. We used a MAF to filter the noise in the pressure data. Subsequently, a threshold-based peak detection algorithm was developed to extract the gait event from the pressure data (Figure 3). The threshold was set to 30 N to reduce noise because the maximum range of the pressure sensor used in this study was 60 N, and most of the noise was lower than 30 N (Figure 4). As the typical walking frequency for a person is 0.5–3 Hz, it can be considered that the time interval of pressure peaks generated by walking is approximately 0.3 s–1 s. Therefore, we set the minimum detection interval of the peak to 0.2 s. The peak intervals less than this period were considered noise.

#### 2.3.3. Sliding Window Algorithm

**Figure 5:**
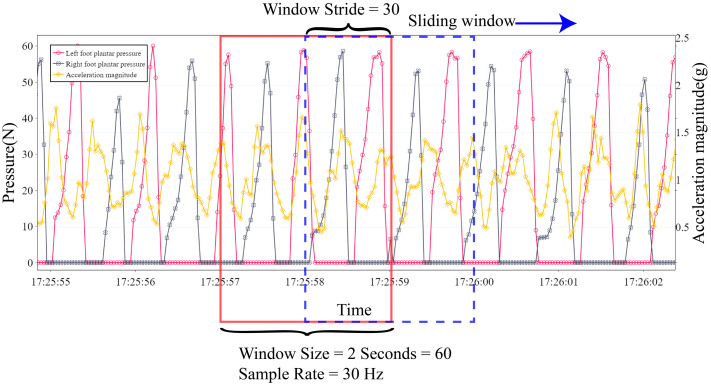
The sliding window algorithm extracts acceleration data in a specific period (window) for input data and the number of pressure data peaks as the label.

The feature extraction of time-series data was performed using the sliding window algorithm with a 50% overlap [34]. Because the human walking frequency is approximately 0.5–3 Hz, the length of the window used in this study was 2 s to contain at least one gait cycle. As shown in Figure 5, data with a sampling rate of 30 Hz contain 60 data points in a 2 s window. If a 50% window stride is applied, half of the data (30 data points) will repeat in the next window. 

### 2.4. Model Building

**Figure 6:**
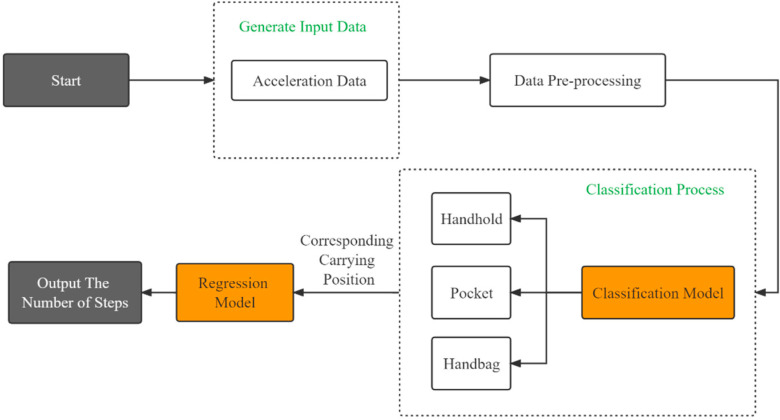
Flow diagram of the walking detection and step-counting algorithm in different carrying positions.

The various carrying positions of a smartphone will result in various acceleration signal patterns [14]. Therefore, it is necessary to use a classification model to classify the carrying position before counting steps. Thus, classification algorithms were used to identify the location of the smartphones, and then the corresponding regression models were applied to the specific carrying position (handheld, pocket, and handbag) to predict the number of steps (Figure 6).

Seven machine learning-based regression and classification algorithms were identified as possible candidates for the step-count algorithm. The main parameters of the algorithms are recorded in Table 1. We used the Keras Python library to create the Multilayer Perceptron and Convolutional Neural Networks, and the Sklearn library to create the Random Forest, Histogram-based Gradient Boost, Support Vector Machine, and K-nearest Neighbors. The Ensemble Model was the weighted average of the Support Vector Machine, Multilayer Perceptron, and Random Forest. Each of the algorithms was evaluated and analyzed, and each of the algorithms was evaluated using the same datasets and data-preprocessing techniques.

## 3. Experimental Evaluation

### 3.1. Experimental Procedure

Data were extracted from six healthy participants (Table 2). We recorded data at a self-paced walking speed (0.8 m/s to 1.5 m/s) for 30 min to train the algorithms and evaluate their performance. The experiment was conducted at Dong-A University, Busan, South Korea. The participants walked along the sidewalk at their preferred gait speed with three smartphones—one placed in the pocket, one held in the hand, and one placed in a handbag. An FSR sensor was placed in the shoe’s sole under the toe, and a mechanical counter was used to record the actual number of steps.

### 3.2. Training Procedure and Testing Procedure

The dataset was divided into a training set and a test set at the ratio of 7:3. In the training procedure, we used the acceleration data corresponding to the three carrying positions to train the classification model, where the input data were the acceleration data, and the label was the corresponding carrying position. Then, the acceleration data and the number of steps obtained from the pressure sensor were used to train the regression model. Each carrying position corresponded to seven proposed regression models, i.e., the three carrying positions corresponded to a total of 3 × 7 regression models.

**Figure 7:**
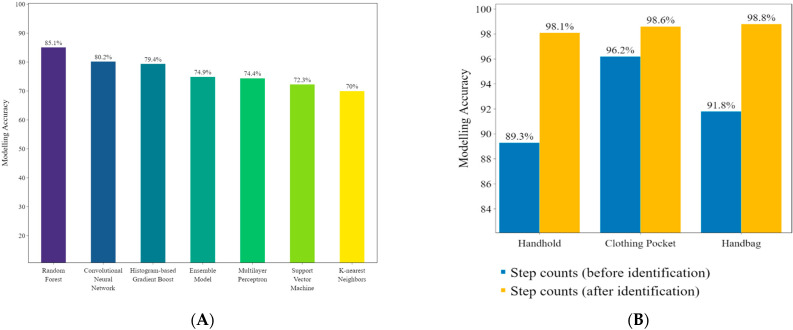
(**A**) Modelling accuracy of each classifier. (**B**) Step-count accuracy before and after position identification (Ensemble Model).

In the testing procedure, the carrying position of the acceleration data was identified by the classification model. Then the number of steps was calculated using the regression model. Figure 7A show the recording of the recognition accuracy of the classification model. Figure 7B shows the comparison of the step-count accuracy before and after position identification.

### 3.3. Accuracy of the Proposed Algorithms

Previous studies [35,36] that used commercial pedometers reported that the step-count algorithm exhibited lower accuracy in low-speed walking compared to high-speed walking. The authors in [36] tested four different step counters at different walking speeds on a treadmill and reported poor performance for all devices at low-speed walking (0.6 m/s). In our study, the data were collected at a low self-paced walking speed (0.8 m/s to 1.5 m/s). In the test set, the accuracy of the Random Forest algorithm reached 85.1%, which was the best overall, which indicates that it had the best modeling accuracy for the various smartphone carrying positions (Figure 7A). These results show that the smartphone carrying position can be accurately identified via accelerometer signals.

The accuracy of the proposed regression algorithms and the commercial smartphone pedometer application was determined as follows:

(3) $$\mathrm{Accuracy}(\%)=(1-\frac{\left|Ne-Nr\right|}{Nr})\times 100\%$$

where *Ne* is the estimated step count, and *Nr* is the real step count.

The accuracy of regression models in Table 3 was obtained after using the classification model to classify the carrying positions (Section 3.2). The commercial pedometer application exhibited lower average accuracy, ranging from 75.3% to 81.9% at self-paced walking speed. In contrast, the Ensemble Model had an average accuracy of 98.5%, which was higher than that of the commercial pedometer application and the other algorithms. A previous study [36] reported a smartphone step-count accuracy of 69.7% in a field test, which was lower than that of the ensemble model obtained in this study. In addition, the six regression algorithms proposed in this study performed better than the commercial pedometer application in all cases.

Our results indicate that different smartphone carrying positions have a significant impact on the step-count accuracy (Figure 7B). Before using the classification algorithm, the accuracy of the ensemble model varied according to the carrying position. In contrast, the proposed Ensemble Model exhibited consistent accuracy after using the classification algorithm to identify the carrying position (98.1% to 98.8%). These results demonstrate that the proposed algorithms are significantly more stable for counting steps than are commercial pedometer applications at varying carrying positions.

## 4. Discussion

In this study, to identify which machine learning algorithm provides the most accurate step-count and carrying position detection, we developed a step-count recognition method using an accelerometer in a practical real-world smartphone use environment.

Our study results indicate that different carrying positions result in different accelerometer signal patterns, which impact the step-count accuracy of the smartphones (Figure 7B). To address this problem, we proposed an ensemble algorithm comprising a classification algorithm and a regression algorithm. The classification algorithm identifies the carrying position of the smartphone and passes this information to the regression algorithm, which then counts the steps accordingly.

The Random Forest algorithm was used to identify the carrying position of the smartphones while walking, with an accuracy of 85.1%. The classification accuracy obtained in this study is higher than that of other acceleration-based solutions. Kaori et al. extracted 60 behavioral characteristics from acceleration data for nine storing positions of a smartphone (neck, pocket, backpack, handbag, and other positions) to build a Support Vector Machine classifier. Their overall accuracy reached 74.6% [37]. In [38], using a polynomial kernel classifier-based Support Vector Machine algorithm, 79.0% classification accuracy was found. The authors pointed out that by using a combination of accelerometer and multispectral sensors, the classification accuracy was increased. However, using multiple sensors significantly increases energy consumption [15]. In another study, the Random Forest algorithm was used to identify the carrying position of the smartphones (hand, bag, pocket) in six daily activity environments (sitting, standing, walking, stairs, running, and bus) [39]. The algorithm achieved an average classification accuracy of 77.3%. In contrast to these studies that focused on identifying the carrying position of smartphones, the Ensemble Model proposed in this study exclusively used the classification algorithm to determine the specific carrying position. The information was then used by the regression algorithm to improve the step-count accuracy.

Previous studies have shown that the accuracy of the step count with smartphones is insufficient under low-speed walking conditions [35,36,40]. Our study also observed similar results (Table 2). After identifying the position of the smartphone, the step-count accuracy of the proposed Ensemble Model was higher than that of the commercial pedometer application and other algorithms. Moreover, the Ensemble Model proposed in this study achieved a higher average step-count accuracy without a pre-set threshold in three carrying positions (clothing pocket, handheld, and handbag). These findings extend those of Vandermeeren, confirming that a machine learning-based step-count algorithm can count steps more accurately than commercial pedometer applications [12]. By identifying the carrying positions of smartphones, the step-count accuracy of the Ensemble Model was 98.5%, which was higher than the accuracy (97.7%) obtained in [12]. In another study, after identifying the carrying positions of smartphones, the step-count accuracy was high and close (no significant changes in different carrying positions) [10]. Although the result was obtained on small datasets (approximately 250 steps in total), our study proves that similar step-count accuracy could be achieved in different carrying positions after identifying the carrying positions (Figure 7B).

To train machine learning-based step-count algorithms and improve their performance, we developed a data acquisition system to collect smartphone acceleration data at a self-paced walking speed. This data acquisition system estimated the time of the gait event by detecting the peak pressure of the plantar pressure data (Figure 4). In this way, the automatic labeling of supervised learning in gait detection was realized, enabling the generation of a large amount of data during the experiment and avoiding the cost and error of manual counting. Future research could involve the training of larger neural network models in this way to achieve higher step-counting accuracies.

Our results indicate that the proposed Ensemble Model achieved higher step-count accuracy and stable performance after identifying the actual carrying position of the smartphones in living conditions. This work will not only promote the research on accelerometer-based walk-counting algorithms based on machine learning technology but also accelerate the development of more applications that rely on accurate step counting.

There are some limitations worth mentioning. Although the proposed algorithm achieves high accuracy in the case of self-paced walking speed (0.8 m/s to 1.5 m/s), participants of varying age ranges and different walking speed experiments could further improve the reliability. Future studies should therefore cover participants of varying age ranges and collect data under different walking speed conditions and carrying positions to obtain higher step-count accuracies and broader applicability.

## 5. Conclusions

In this study, we developed a carrying position-independent step-count algorithm for smartphones. A data collection system was developed to record smartphone acceleration data and plantar pressure data while walking. The proposed data-collection system and step-counting algorithm can be used to collect plantar pressure data and smartphone acceleration data in complex walking environments to provide labels for analyzing gait events from smartphone acceleration data (walking, running, and climbing). On the other hand, by analyzing the plantar pressure data and smartphone acceleration data of patients with gait disorders, the gait disorders can be observed. The number of steps comprises the basic data for many applications (such as walking speed and walking distance). Therefore, the proposed step-counting algorithm can be considered packaged as a smartphone pedometer application, or it can be built into other health monitoring applications.

Furthermore, our study revealed the potential to improve the performance of smartphone pedometers by plantar pressure data and indicated that the proposed ensemble algorithm approaches provide higher accuracy, ranging from 98.1% to 98.8%, at self-paced walking speeds. Therefore, machine learning-based ensemble algorithms can count effectively and accurately predict step counts under different smart phone carrying positions. Future research could use this method in larger, more diverse samples to identify a higher number of more differentiated gait patterns and plantar pressure, for example, collecting the plantar pressure and smartphone acceleration data of people of different age groups when walking differently to develop a step-counting algorithm suitable for people.

## Figures and Tables

**Table 1:** Parameters of proposed algorithms.

Classifiers	Parameters	Regressors	Parameters
Multilayer Perceptron	Hidden layers = 4 Total units = 920	Multilayer Perceptron	Hidden layers = 4 Total units = 920
Convolutional Neural Networks	1D convolutional layers = 2 Max pooling layers = 2 Fully connected layers = 1	Convolutional Neural Networks	1D convolutional layers = 4 Max pooling layers = 4 Fully connected layers = 2
Random Forest	N_estimators = 200 Max_depth = 200	Random Forest	N_estimators = 500
Histogram-based Gradient Boost	Default	Histogram-based Gradient Boost	Default
Support Vector Machine	Kernal = linear	Support Vector Machine	Kernal = linear
K-nearest Neighbors	Default	K-nearest Neighbors	Default
Ensemble Model	-	Ensemble Model	-

**Table 2:** Demographic characteristics of the participants.

No.	Gender	Age (Years)	Weight (kg)	Height (cm)	Steps	Time (Minutes)
1	Male	27	49.5	170.0	2451	30
2	Male	26	68.3	180.0	3100	30
3	Male	27	74.5	170.0	3122	30
4	Male	29	84.2	177.5	2850	30
5	Female	26	41.3	160.0	2431	30
6	Female	27	69.1	170.5	3025	30

**Table 3:** Accuracies of step-count algorithms.

	Mean Accuracies (%)								
Carrying Position	Regression Algorithms							Pedometer Application	Average
	Random Forest	Convolutional Neural Network	Histogram-Based Gradient Boost	Multilayer Perceptron	Support Vector Machine	K-Nearest Neighbors	Ensemble Model	Rakuraku Smartphone Pedometer	
Handheld	94.1	90.4	87.7	90.8	79.4	82.1	98.1	75.3	87.2
Pocket	85.0	91.3	95.8	99.3	89.8	95.7	98.6	80.8	92.0
Handbag	89.2	89.0	86.9	91.6	77.4	83.6	98.8	81.9	87.3
Average	89.4	90.2	90.2	93.9	82.2	87.1	98.5	79.3	

## Data Availability

Qualified researchers can obtain the data from the corresponding author (htpark@dau.ac.kr). The data are not publicly available due to privacy concerns imposed by the IRB.
